# Circular of the Surgeon General

**Published:** 1845-08-01

**Authors:** 


*Circular of the Surgeon General.*—The Surgeon general of the U. S. A. has issued a circular to the officers of the medical staff, directing them to make trial of *Salicine* (the active principle of the bark of the common *willow*) which has been recommended for its *febrifuge* and *anti-periodic* virtues, and to report their observations, on or before the expiration of the current year. He intimates that the results will be given to the profession.

